# *In Vitro* Antioxidant, Anti-Inflammatory and Skin Permeation of *Myrsine africana* and Its Isolated Compound Myrsinoside B

**DOI:** 10.3389/fphar.2019.01410

**Published:** 2020-01-08

**Authors:** Bianca Fibrich, Xinyi Gao, Ashana Puri, Ajay K. Banga, Namrita Lall

**Affiliations:** ^1^ Department of Plant and Soil Sciences, University of Pretoria, Pretoria, South Africa; ^2^ Center for Drug Delivery Research, Department of Pharmaceutical Sciences, College of Pharmacy, Mercer University, Atlanta, GA, United States; ^3^ School of Natural Resources, University of Missouri, Columbia, MO, United States

**Keywords:** *Myrsine africana*, myrsinoside B, lipoxygenase, skin delivery, maltose microneedles

## Abstract

Dermal aging is characterized by states of oxidative stress, chronic inflammation, and abnormal proteolytic degradation due to the action of hydrogen peroxide, superoxide, 5-lipoxygenase, and elastase, respectively. Noteworthy elastase inhibition has previously been reported, and so this study aimed to investigate the ability of *Myrsine africana* and myrsinoside B to reduce the activity of hydrogen peroxide, superoxide, and 5-lipoxygenase as supplementary mechanisms of action by which *M. africana* may reduce the appearance of wrinkles. The use of maltose microneedles were also investigated as a means to enhance the delivery of myrsinoside B into the skin as this is a crucial aspect to investigate when characterizing the efficacy of an active ingredient. *Myrsine africana* has traditionally been used for skin allergies, boils, and to purify blood (as an astringent) and was selected for this study based on it use in skincare. The crude extract exhibited IC_50_’s of 56.08 ± 2.88 and 132.74 ± 1.64 µg/ml against the hydrogen peroxide and superoxide radicals, while myrsinoside B exhibited IC_50_’s of 52.19 ± 4.16 and 192.14 ± 3.52 µg/ml, respectively. The IC_50_ of the extract and compound against 5-lipoxygenase was 29.65 ± 2.92 and 29.33 ± 3.08 µg/ml, respectively. No toxicity was observed *in vitro* at the highest concentration tested. Microneedle treatment increased the permeation of the active through the skin after 24 h to 12.46 ± 5.14 µg/cm^2^ compared to the passive group (1.30± 0.85 µg/cm^2^). The amount of active retained in the epidermis and dermis was 8.97 ± 0.90 and 6.98 ± 0.73 µg/cm^2^ respectively, greater than the retention observed in the passive group (3.24 ± 1.41 and 3.27 ± 1.47 µg/cm^2^, respectively). *M. africana* and myrsinoside B showed promising antioxidant and anti-inflammatory activity thus supporting the potential of *M. africana* and myrsinoside B as anti-wrinkle agents. Further, treatment of dermatomed human skin with maltose microneedles facilitated topical delivery of myrsinoside B and provided an effective means for compound delivery to ensure maximum effect.

## Introduction

The science of aging skin has long been a topic of interest with the demand for effective botanical actives a particular area of curiosity. Dermal aging may arise from either intrinsic or extrinsic factors and is characterized by states of oxidative stress, abnormal degradation of structural components housed within the dermis, and chronic inflammation. Specifically, hydrogen peroxide and superoxide are produced in this process and interact undesirably with biological molecules to enhance the activity of proteases such as elastase which proceed to degrade structural components. Simultaneously, inflammatory enzymes such as 5-lipoxygenase are recruited to the affected site and further elicit a stress response. Coupled to this, reduced cellular turnover rates, abridged production of structural components, and the naturally diminishing capacity of the skin to maintain homeostasis contribute synergistically to the formation of wrinkles (Pillai et al., 2005; Pandel et al., 2013; Peng et al., 2014; Ryu et al., 2014).

The ability of biologically active molecules to penetrate the skin is equally significant to ensure delivery to the targeted site. Microneedles comprise a commonly used physical enhancement technology for topical and transdermal delivery of hydrophilic drugs. These work by creating aqueous micron-sized channels in the skin that enable transportation of active constituents into the skin or systemic circulation (Chen et al., 2008). The use of microneedles in cosmetics is promising, however, the therapeutic efficacy of microneedle-based products is still under investigation in clinical trials. Microneedles are actively employed in the cosmetic industry and are even available for home use, making their potential particularly promising (Bhatnagar et al., 2017).

*Myrsine africana* L. (Primulaceae) is a shrub commonly known as the African Boxwood. It is found throughout Asia, Macaronesia, and Africa and has a wealth of traditional knowledge alluding to its applications. Traditionally, a leaf decoction is prepared for the treatment of acne, pigmentation disorders, wound healing, cellulitis, and as a blood purifier by the Southern Sotho, Kwena, and Tswana tribes of South Africa. Similarly, a leaf decoction is prepared for the treatment of skin allergies and as a blood purifier in Pakistan. In Kenya, people of the Samburu district prepare a decoction from ground seeds for the treatment of wounds. Chinese folklore also indicates that it is traditionally used for the treatment of diarrhea, pulmonery tuberculosis, rheumatism, toothache, and hemorrhaging (Kang et al., 2007). It is also used in various culinary applications as a fragrance, flavoring agent, spice, and appetizer (Ahmad et al., 2011b).Additional uses include as an anthelmintic, antibacterial, for the treatment of boils, and scanty urine (Watt and Breyer-Brandwijk, 1962; Killick, 1969; Githiori et al., 2002; Abbasi et al., 2010). The reported use of *M. africana* as a blood purifier spurs interest around its potential application as an anti-wrinkle agent as such agents often play an important role in maintaining healthy skin (Kishore et al., 2018).

Previous studies have identified and characterised myrsinoside B (94.39% purity) amongst other compounds (Kishore et al., 2018). Lall et al. (2017) reported noteworthy elastase inhibition for *M. africana* (28.04 µg/ml) and myrsinoside B (4.69 µg/ml). The aim of this study was therefore extrapolate on the anti-aging activity of *M. africana* and myrsinoside B by determining the ability of the crude extract and compound to respectively scavenge hydrogen peroxide and superoxide radicals, and inhibit 5-lipoxygenase. Further, this study aimed to characterize and identify a means of delivery of myrsinoside B into the skin. The extract and isolated myrsinoside B samples used in this study were the same as that produced in previous studies (Lall et al., 2017; Kishore et al., 2018).

## Materials

Ethanol, methanol (M), ethyl-acetate (EA) and hydrogen peroxide were purchased from Associated Chemical Enterprises Chemicals (Southdale, South Africa). XTT cell proliferation kit II, actinomycin D, soybean lipoxygenase (type V), linoleic acid, xylenol orange, ferrous sulphate, nitroblue tetrazolium chloride, dimethyl sulfoxide (DMSO), propylene glycol (PG) and 10X Phosphate-buffered saline, pH 7.4 (PBS) were obtained from Sigma-Aldrich (St Louis, MO, USA). The HaCat cell line was purchased from Highveld Biological (Pty) Ltd. (South Africa). Dulbecco’s Modified Eagle’s Medium (DMEM), trypsin-EDTA, fetal bovine serum (FBS), phosphate buffer saline (PBS), and antibiotics were supplied by Thermofisher scientific (Modderfontein, Johannesburg, South Africa). Citric acid, disodium hydrogen phosphate, triethanolamine (TEA) and formic acid were obtained from Fisher Scientific (Fair Lawn, NJ, USA). Carbopol 980NF was procured from Lubrizol Corporation (Wickliffe, Ohio, USA). Dermatomed human cadaver skin was purchased from the New York Fire Fighter (NY, USA). All other reagents were of analytical grade.

## Methods

### Plant Collection and Extract Preparation

*M. africana* was collected in 2011 from the Manie van der Schijff Botanical Garden in Pretoria, South Africa (S25° 45’ 21” E28° 13’ 51”) and identified by Ms. Magda Nel at the H.G.W.J. Schweickerdt Herbarium (PRU) where a voucher specimen (MA-S-2013-1) was deposited. The preparation of a crude ethanolic extract and isolation of myrsinoside B were exactly as previously reported (Lall et al., 2017; Kishore et al., 2018).

### Hydrogen Peroxide Scavenging

Hydrogen peroxide scavenging potential was determined according to the method of Kumar et al. (2012). Briefly, the ethanolic extract of *M. africana* and the positive drug control, L-ascorbic acid, were prepared to stock concentrations of 10,000 µg/ml and serially diluted in Eppendorf tubes to yield a concentration range of 400–3.125 µg/ml. To this, hydrogen peroxide (10 mM) prepared in potassium phosphate buffer (pH 7, 50 mM) was added and the reaction mixture incubated at room temperature in the dark. The FOX reagent was prepared using sulfuric acid (0.025 M), D-sorbitol (0.1 M), xylenol orange (1 x 10^-3^ M), ferrous sulphate (250 mM), and methanol:water 9:1, and added to the reaction mixture which was incubated for a further 20 min in the dark at room temperature. The Eppendorf tubes were then centrifuged for 1 min to remove flocculation and the supernatant removed. The absorbance of the supernatant was measured at 580 nm using a BIO-TEK Power-Wave XS multi-well plate reader (A.D.P, Weltevreden Park, South Africa) and KC Junior software. The IC_50_ values were calculated using GraphPad Prism 4 software.

### Superoxide Radical Scavenging

The alkaline DMSO method of Kumar et al. (2012) was used to determine the superoxide radical scavenging potential of *M. africana* and myrsinoside B. These samples and the positive control, Quercetin, were prepared to stock concentrations in DMSO and serially diluted to yield a range of concentrations (400–3.125 µg/ml). Alkaline DMSO was prepared by dissolving sodium hydroxide in DMSO (0.2 mg/ml). In a 96-well microtiter plate, alkaline DMSO was added to the plant extract of *M. africana*, myrsinoside B and quercetin. A color control, negative control, and vehicle control containing DMSO were included. Nitro blue tetrazolium (NBT) chloride was dissolved in DMSO at a 1:1 ratio and this was added to the test samples, including the vehicle control and the plates were read immediately at 560 nm using a BIO-TEK Power-Wave XS multi-well plate reader (A.D.P, Weltevreden Park, South Africa) and KC Junior software. The IC_50_ values were calculated using GraphPad Prism 4 software.

### 5-Lipoxygenase Inhibition

The 5-LOX inhibition was investigated according to the method of Chung et al. (2009) in which lipoxygenase from soybean (type V) (100 ng/ml) prepared in Tris-HCl (pH 7.4, 50 mM) was incubated for 5 min at room temperature with the test samples and positive control respectively. All test samples were prepared to stock concentrations of 20 mg/ml in DMSO and serially diluted to a concentration range of 400–3.125 ug/ml. A vehicle control containing 0.2% DMSO, a blank containing 5-LOX to which linoleic acid was added after the addition of the FOX reagent, positive control containing caffeic acid at the same concentration range as the extracts, and color controls containing the plant extract and Tris-HCl buffer were included. The reaction was then initiated by the addition of linoleic acid (140 μM) prepared in Tris-HCl (pH 7.4, 50 mM) and incubated for 20 min at room temperature in the dark. The reaction was terminated through the addition of freshly prepared FOX reagent (30 mM sulfuric acid; 100 µM xylenol orange; 100 µM ferrous sulphate, and methanol: water (9:1). The formation of a color complex was allowed to develop for 30 min at room temperature, and the absorbance was measured at 560 nm using a BIO-TEK Power-Wave XS multi-well plate reader (A.D.P, Weltevreden Park, South Africa) and KC Junior software. The IC_50_ values were calculated using GraphPad Prism 4 software.

### Cell Culture and Cytotoxicity

Human keratinocytes were maintained in Dulbecco’s Modified Eagle’s Medium supplemented with 10% Fetal Bovine Serum and 1% antibiotics (100 U/ml penicillin, 100 µg/ml streptomycin, and 250 µg/ml fungizone). The cells were grown statically at 37°C in a humidified incubator set at 5% CO_2_“CO2:Carbon dioxide” . Once confluent, the cells were sub-cultured by treating them with trypsin-EDTA (0.25% trypsin containing 0.01% EDTA) for a maximum of 10 min. The cytotoxicity was evaluated using the XTT cell proliferation Kit II according to the method of Zheng et al. (2001). Cells (1 x 10^5^ cells/ml) were seeded in a 96-well microtitre plate and allowed to attach for 24 h at 37°C and 5% CO_2_. *M. africana* and myrsinoside B were prepared to stock concentrations of 20 mg/ml DMSO and the cells were treated at concentrations ranging from 400–3.13 µg/ml for test samples and concentrations ranging between 0.5 and 0.002 µg/ml for the positive drug control, Actinomycin D. A vehicle control was included where cells were treated with 2% DMSO. The treated cells were further incubated for 72 h followed by the addition of 50 µl XTT to a final concentration of 0.3 mg/ml. The plates were incubated with the viability reagent for 2 h and the absorbance of the color complex measured at 490 nm with a reference wavelength set at 690 nm for XTT using KC Junior software and a BIO-TEK Power-Wave XS multi-well plate reader (A.D.P, Weltevreden Park, South Africa). The IC_50_ values were calculated using GraphPad Prism 4 software.

### Determination of Myrsinoside B Concentration in the *Myrsine africana* Extract and Formulation Into a Hydrogel

The concentration of myrsinoside B in the *M. africana* extract was determined by preparing stock concentrations of the extract (10mg/ml, 24.5 mg/ml, and 45 mg/ml) in propylene glycol. The concentrations were agitated at 150 rpm overnight and centrifuged at 13,400 rpm for 10 min to remove undissolved constituents. The suprnatant of each concentration was then dissolved in 10 ml PBS and filtered using a 0.22 µm syringe (Cell treat Scientific Products, Shirley, MA, USA). The concentration of myrsinoside B was determined using HPLC. For the formulation of the hydrogel, *M. africana* was dissolved in propylene glycol according to the method of Gao (2018). The agitated at 150 rpm overnight. The mixture was centrifuged at 13,400 rpm for 30 min to remove undissolved constituents and filtered through a 0.45 µm syringe filter followed by a 0.22 µm syringe filters and collected in a scintillation vial. A hydrogel was prepared by adding Carbopol 980 NF (1% w/v) to the solution slowly and stirred using a magnetic stirrer overnight at room temperature (VWR, Radnor, PA, USA). The mixture was neutralized with TEA to form a transparent gel matrix with a pH of approximately 5.5. The concentration of myrsinoside B in the hydrogel formulation was determined by sampling 10 µl of the formulation from three different locations in the container. These samples were diluted 200 times in PBS and injected for HPLC analysis.

### Microneedle Treatment and Confocal Microscopy

Eighty one sharp tipped maltose microneedles (500 µm in length) arranged in three parallell rows were used to physically enhance the delivery of myrsinoside B into dermatomed human skin. Parafilm was used to support the skin. The malstose microneedles were injected vertically into the skin for one minute and removed. The depth of the microchannels created by the microneedle injection was measured using a computerized Leica SP8 confocal laser scanning microscope (Leica Microsystems, Heerbrugg, Switzerland) with the 10X objective. Fluoresoft^®^ (0.35%) solution was applied to the treatment site for one minute and thoroughly wiped off using kimwipes and alcohol swabs. The skin sample was scanned under the confocal microscope at an excitation wavelength of 496 nm. X-Z sectioning was used to estimate the depth of the microchannels by capturing a series of images at the same location but from different depths (from the skin surface to the point where no fluorescent signal was observed) (Puri et al., 2016).

### *In Vitro* Skin Permeation, Skin Extraction and HPLC Analysis

The permeation of myrsinoside B through dermatomed human skin was studied *in vitro* using static vertical Franz diffusion cells (n ≥ 3). The temperature of the receptor compartment was set at 37°C using a circulating water bath to maintain the temperature of the surface of the skin at 32°C. Non-treated and maltose microneedle treated skin was mounted on the Franz cells with the stratum corneum facing up. The *M. africana* extract hydrogel (200 µl) was applied in the donor chamber, and the receptor chamber was filled with 5 ml 1 X PBS (pH7.4 buffer). The receptor buffer (300 µl) was withdrawn at pre-determined time points and replaced with 300 µl fresh buffer solution. Samples were filtered using a 0.22 µm syringe filter and analyzed using HPLC. After the 24 h permeation study, the remaining hydrogel in the donor compartment was removed using Q tips and the surface of the skin cleaned properly. The epidermis was removed from the skin using forceps and minced. The underlying dermis was cut into small pieces using scissors. The epidermis and dermis samples were transferred into a 6-well plate to which 2 ml PBS was added and maintained on a shaker (New Brunswick Scientific Co. Inc., Edison, NJ, USA) at 150 rpm for 4 h. The samples were then filtered through 0.22 µm syringe filter (Celltreat Scientific Products, Shirley, MA, USA) and analyzed using HPLC using Waters Alliance HPLC system (e2695 separation module) (Waters Co., Milford, MA, USA) coupled to a 2996 photodiode array detector (Waters Co., Milford, MA, USA). Reverse-phase high-performance liquid chromatography (RP-HPLC) was used to determine the levels of myrsinoside B. A Kinetex Luna Phenyl-Hexyl (150 x 4.6 mm, 2.6µm) column was used at room temperature with methanol and 0.01% v/v formic acid in deionised water (30:70% v/v) as the mobile phase. A sample volume of 30 µl was injected at a flow rate of 0.5 ml/min and analyzed at the detection wavelength of 278 nm.

### Data Analysis

The cytotoxicity, hydrogen peroxide scavenging, superoxide scavenging, and 5-lipoxygenase inhibitory results are depicted as the mean ± SD (n = 3). Statistical analysis was done using one-way analysis of variance (ANOVA) followed by Tukey’s multiple comparison test using the GraphPad Prism statistical software to determine the statistical differences observed. All statistical evaluations for the results from the permeation studies were performed using Student’s t-test and p-value < 0.05 was considered for concluding significant difference (n ≥3).

## Results

### *In Vitro* Biological Activity

The results for the in vitro biological activity investigated are summarized in **Table 1**.

**Table 1:** The *in vitro* cytotoxicity, 5-lipoxygenase inhibitory, hydrogen peroxide and superoxide scavenging activity of the ethanolic extract of *Myrsine africana* and its isolated compound, myrsinoside B.

	H_2_O_2_^a^ scavenging (IC_50_^e^ in µg/ml ± SD^l^)	O_2_^-b^ scavenging (IC_50_^e^ in µg/ml ± SD^l^)	5-LOX^c^ inhibition (IC_50_^f^ in µg/ml ± SD^l^)	Cytotoxicity^d^ (IC_50_^g^ in µg/ml ± SD^l^)
*Myrsine africana* ethanolic extract	56.08 ± 2.88^A^	132.74 ± 1.64^A^	29.65 ± 2.92^A^	>400
Myrsinoside B	52.19 ± 4.16^A^	192.14 ± 3.52^B^	29.23 ± 3.08^A^	>400
Positive drug control	47.62 ± 3.34^h,B^	15.29 ± 3.68^i,C^	14.87 ± 0.69^j,^ ^B^	<5 x 10^-3^ ^k^

Hydrogen peroxide^a^, Superoxide^b^, 5-Lipoxygenase^c^, Tested against Human Keratinocytes^d^, Fifty percent scavenging concentratione, Fifty percent inhibitory concentration^f^, Concentration at which fifty percent of the cell population remains viable^g^, L-Ascorbic Acid^h^, Quercetin^i^, Caffeic acid^j^, Actinomycin D^k^. Values are expressed as mean ± SD (n = 3)^l^. Statistical analysis was done using one-way analysis of variance (ANOVA) with Tukey’s multiple comparison test, where A, B and C represent significant differences (p < 0.01).

### Hydrogen Peroxide and Superoxide Radical Scavenging

*M. africana* scavenged 50% of the hydrogen peroxide free radical at a concentration of 56.08 ± 2.88 µg/ml, not significantly different from myrsinoside B which exhibited an IC_50_ of 52.19 ± 4.16 µg/ml. Both of these were found to be significantly higher (p < 0.01) than the positive control, L-ascorbic acid which exhibited an IC_50_ of 47.62 ± 3.34 µg/ml. The *M. africana* ethanolic extract scavenged 50% of the superoxide radical at a concentration of 132.74 ± 1.64 µg/ml, significantly lower than the IC_50_ of Myrsinoside B (192.14± 3.34 µg/ml), but significantly higher than the positive drug control quercetin (15.29 ± 3.68 µg/ml) (p < 0.01) (**Table 1**).

### 5-LOX Inhibition

The results indicate that both the crude extract of *M. africana* and myrsinoside B showed inhibition of 5-LOX, with IC_50_’s of 29.65 ± 2.92 and 29.23 ± 3.08 µg/ml respectively, not significantly different from one another, but significantly higher (p < 0.01) than caffeic acid (14.87 ± 0.69 µg/ml) (**Table 1**).

### *In Vitro* Cytotoxicity

The *in vitro* cytotoxicity investigation on human keratinocytes showed the ethanolic extract of *M. africana*, as well as myrsinoside B to be non-toxic to human keratinocytes *in vitro* at the highest concentration tested (400 µg/ml). The positive control, Actinomycin D, exhibited an IC_50_ of < 5 x 10^-3^ μg/ml, lower than the lowest concentration investigated (**Table 1**).

### Myrsinoside B Concentration in *M. africana* Extract and Formulation Into a Hydrogel

Different amounts of the *M. africana* extract (10, 24.5 and, 45 mg) were added to 1 ml of PG to determine the content of myrsinoside B in the extract. For each concentration, the test was performed in triplicate. The average concentration of myrsinoside B in *M. africana* extract was found to be 26.99 ± 0.18%. Once *M. africana* was formulated into a hydrogel for the permeation study, three locations within the hydrogel were sampled and the average concentration of myrsinoside was found to be 5.06 ± 0.12 mg/ml (**Supplementary Data Sheet 1**).

### Depth of Microchannels Created by Maltose Microneedles

**Figure 1:**
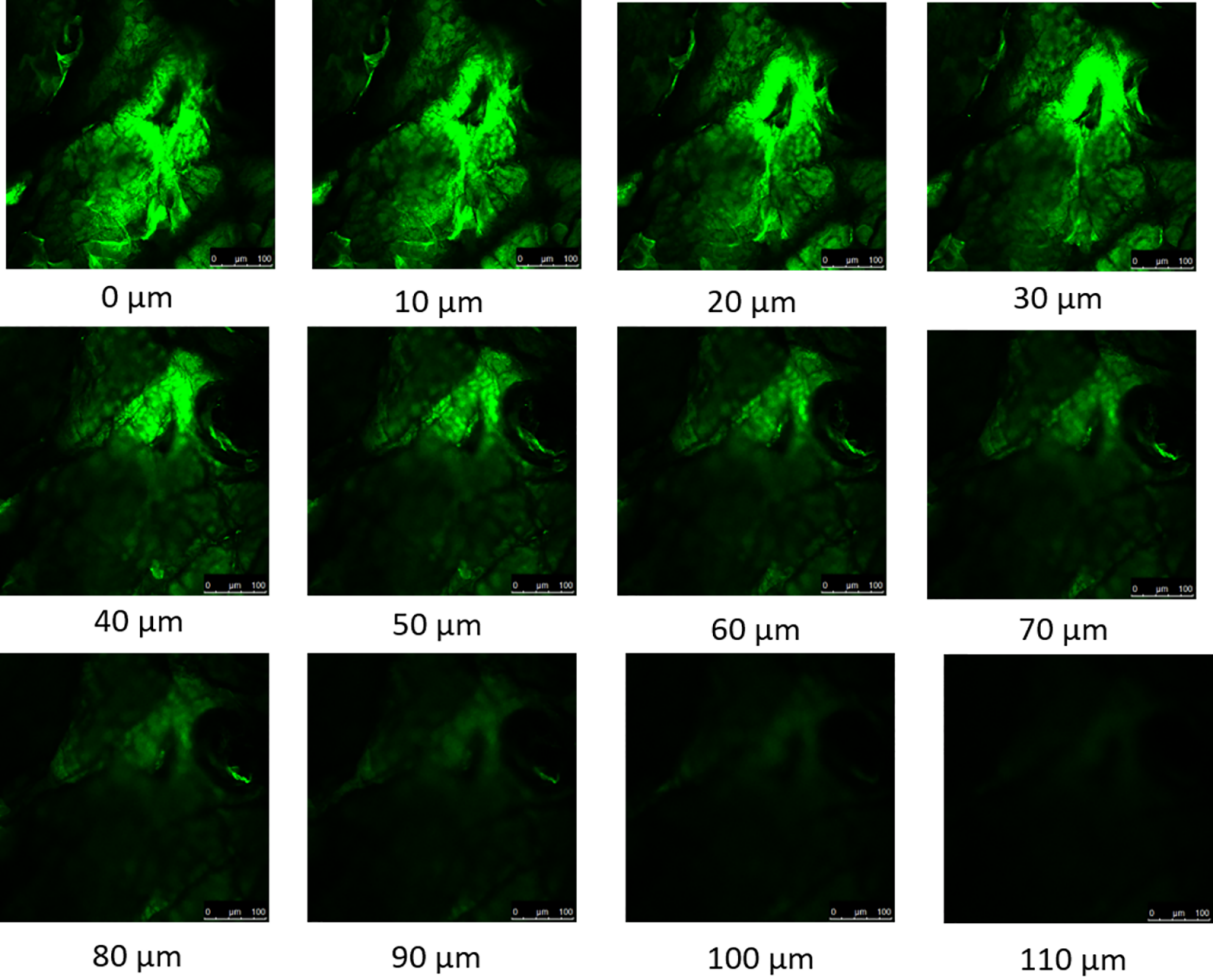
Confocal microscopy z-stack of micropores created by maltose microneedle reveals the depth of penetration to be approximately 110 µm.

The depth of the micropores were estimated using confocal microscopy z-stack which captured a sequence of the images at the same horizontal position (x, y) at different depths (z). The results (**Figure 1**) indicated that the microchannels created by maltose microneedles in dermatomed human skin were approximately 110 µm deep.

### *In Vitro* Permeation and Skin Extraction Studies

**Figure 2:**
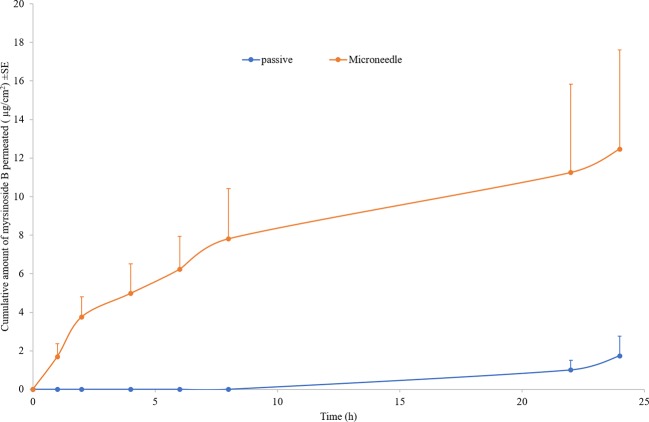
Cumulative quantities of permeated myrsinoside B for the maltose microneedle treated skin compared to passive delivery (µg/cm^2^ + SE, n ≥ 3). Maltose microneedle treatment significantly increases the quantity of myrsinoside B permeated and reduces lag time (n ≥ 3, Student’s t-test p < 0.05 indicates significant difference).

**Figure 3:**
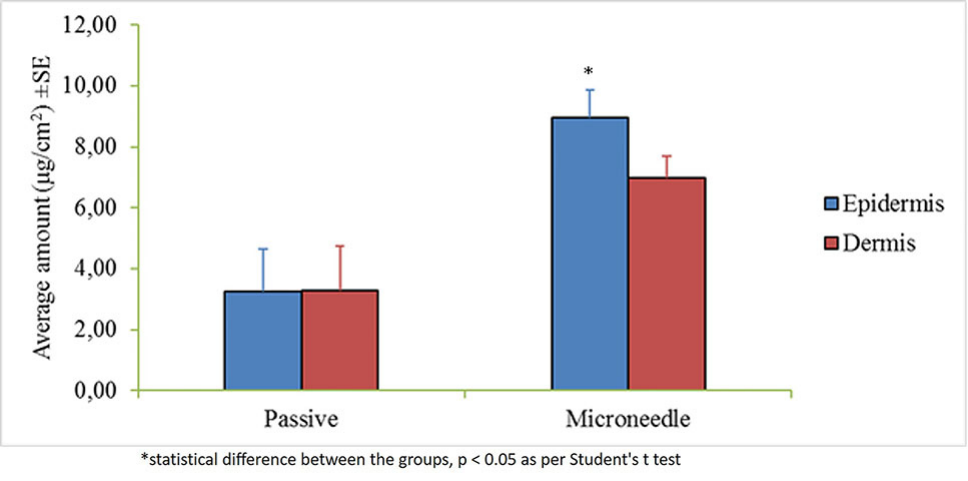
Average amount of myrsinoside B retained in the epiderms and dermis of the human dermatomed skin (µg/cm^2^ + SE). The epidermis of the maltose microneedle treated group retained significantly greater amounts of myrsinoside B compared to the epidermis of the passive delivery group, however, no statistical difference was observed for the dermis (n ≥ 3, Student’s t-test p < 0.05 indicates significant difference).

The control permeation study indicated low passive permeability of myrsinoside B after 24 h (1.30 ± 0.85 µg/cm^2^) due to its hydrophilic nature. Maltose microneedle treatment significantly increased the permeation of myrsinoside B to 12.46 ± 5.14 µg/cm^2^ (p < 0.05) (**Figure 2**). The lag time for permeation was also significantly reduced to 1 h, compared to the passive group (more than 10 h). The drug amount retained in the epidermis after 24 h for the microneedle treatment group was observed to be 8.97 ± 0.90 µg/cm^2^, significantly greater than in the control group (3.24 ± 1.41 µg/cm^2^, p < 0.05). However, the drug amount in dermis layer for microneedle group (6.98 ± 0.73 µg/cm^2^) was not significantly different than the control group (3.27 ± 1.47 µg/cm^2^, p > 0.05) (**Figure 3**).

## Discussion

Three conditions of the skin make it especially vulnerable to the effects of reactive oxygen species; firstly, it is exposed to high doses of UV radiation often. Secondly, it has a high oxygen tension, and thirdly, it is rich in unsaturated fatty acids. Phagocytic and polymorphonuclear cells, such as neutrophils and monocytes, produce superoxide, molecular oxygen, hydroxyl radicals, and hydrogen peroxide in response to inflammation. Furthermore, infiltrating leukocytes also expose the skin to a respiratory burst, producing elevated levels of superoxide. During this process, catalytic iron is also released from ferritin which causes additional damage. The effects of the hydroxyl radical include damaging biological macromolecules and releasing cellular components, ultimately leading to cell death. In addition, ROS activate transcription factors such as AP-1 and NFκβ, which are regulated by cellular redox levels, to further stimulate the aging process (Peng et al., 2014). Antioxidant investigations have been conducted on various extracts of *M. africana*, which suggest that *M. africana* holds a higher affinity for scavenging superoxide and hydrogen peroxide than alternative radicals such as nitric oxide (Ahmad et al., 2011b). Gul et al. (2017) investigated the antioxidant propensity of various extracts of *M. africana* including an ethanolic extract, using hydrogen peroxide, and superoxide radicals amongst others (DPPH and ABTS). From their study, the findings indicate the ethanolic extract of *M. africana* to have an IC_50_ of 46.61 ± 0.08 µg/ml against the hydrogen peroxide free radical and 122.64 ± 1.55 µg/ml against the superoxide free radical, which supports the findings of the current study. Further investigations into the phytochemistry of *M. africana* revealed the presence of a range of secondary metabolites including flavonoids and polyphenols, which may contribute to the antioxidant activity observed in both studies (Gul et al., 2017). By inactivating specific free radicals such as hydrogen peroxide and superoxide, the initiation of the aging cascade is diminished and the states of oxidative stress which continue to fuel the process reduced.

Inflammation is a complex biological response to external stimuli such as UV-radiation, pathogens, and irritants. Compromised cell membranes resulting from this damage release arachidonic acid that is then processed by either the cyclooxygenase or lipoxygenase pathway. The COX pathway involves two isoforms of the cyclooxygenase enzyme which act to produce thromboxane’s and prostaglandins, while the lipoxygenase pathway produces a range of leukotrienes. Of these leukotrienes, leukotriene B4 (LTB4) has been noted to produce the most pronounced effect in the inflammatory response and is produced predominantly by 5-LOX. By diminishing the inflammatory response, the production of reactive oxygen species and free radicals is curbed, reducing activation of cellular components which are mediated by the cellular redox potential (Pillai et al., 2005). Thus, the inflammatory response extends to the processes involved with oxidative stress and abnormal proteolytic degradation, to form an interlinked network that is collectively targeted. A study by Ahmad et al. (2011a) investigated a crude methanolic extract and various fractions of the aerial parts of *M. africana* for their anti-inflammatory activity in a phagocyte induced human neutrophil model which measures the production of reactive oxygen species. The ethyl acetate and n-hexane fractions of the plant showed the highest percentage inhibition at 500 µg/ml (44.8 and 40.9%, respectively). The data in the current study indicates the extract of *M. africana* may be a potential anti-inflammatory agent with a more specific mechanism of action towards inflammatory enzymes such as lipoxygenase, but future studies should investigate additional inflammatory enzymes such as cyclooxygenase-II. By targeting both classes of inflammatory enzymes the manifestation of inflammation will be more significantly reduced.

Edible plants, culinary plants and spices are considered highly toxic when exhibiting an IC_50_ lower than 50μg/ml, moderately toxic 50–200μg/ml, low toxicity between 200–1,000μg/ml, and non-toxic >1,000μg/ml (Kuete and Efferth, 2015). Low levels of toxicity as observed in the present study were similarly found in a study by Gul et al. (2017) as well as irritancy studies in a clinical trial setting in an investigation by Lall et al. (2017).

As the largest organ of the human body, the most important function of the skin is to protect the underlying tissues and internal organs from external stimuli. This barrier function is achieved mainly by the topmost layer of the skin, stratum corneum, which is composed of layers of dead, flattened, keratin-filled cells and has lipophilic nature. For most hydrophilic drugs, like myrsinoside B, passive permeation efficiency is usually very low as observed (Log P -0.69), and enhancement techniques are needed to disrupt the skin barrier and facilitate delivery. The microneedle technique has been found to facilitate many molecules through skin previously by creating micro-sized channels through stratum corneum and epidermis. Microneedles are well investigated and have been demonstrated to significantly enhance the intradermal delivery of recombinant HIV-1 CN54gp, nile red-loaded nano-particles, 5-aminolevulinic acid, DNA vaccine, botulinum toxin A, nano-encapsulated rhodamine B dye, and epigallocatechin-3-gallate (Nguyen and Banga, 2015; Puri et al., 2017). In our study, the depth of the microchannels created by the maltose microneedles was found to be around 110 nm, indicating the successful distruption of the stratum corneum and epidermis without reaching deep dermis nerves which stimulate pain. A significantly higher amount of myrsinoside B permeated the aqueous channels created by the microneedles than passive application on intact skin, which continued to accumulate over the 24 h period at a greater rate than passive application. More importantly, the lag time for skin permeation of the active was dramatically shortened by microneedles. By delivering myrsinoside B directly into the skin the efficacy of the active is enhanced. The slow rate of penetration and accumulation observed in the passive application suggests that the majority of the active is not absorbed and will ultimately be lost through sweating or touching the skin. Although the quantity of myrsinoside B retained within the dermis and epidermis is at concentration higher than its reported elastase inhibitory activity as reported by Lall et al. (2017), it is lower than the active concentrations obtained in the present study, suggesting that additional means to improve the delivery of myrsinoside B should be investigated. When comparing the amount of myrsinoside B in the dermis and epidermis, only the epidermis of the microneedle treated skin showed significantly higher amounts of myrsinoside B. The dermis of the microneedle treated skin retained less myrsinoside B than the epidermis, suggesting a barrier preventing the efficient transport of myrsinoside B across the dermal-epidermal junction. Future investigations should focus on the delivery of myrsinoside B to the dermis where its efficacy would be most pronounced, which may be achieved by increasing the length of the microneedle channels to penetrate deeper into the epidermis.

The results indicate *M. africana* and myrsinoside B are capable of targeting numerous facets of the aging pathway, however, investigating additional major targets would further characterize any additional mechanisms of action by which this plant extract and its isolated compound may exert their action. While significant differences were observed in the superoxide scavenging investigation, the activity profiles of *M. africana* and myrsinoside B were similar for the other investigations, and suggest that the whole extract or a semi-pure fraction can be used for anti-aging therapies as a cost-effective alternative to compound isolation. Myrsinoside B could be used as a biomarker. Clinical studies by Lall et al. (2017) indicate even with passive application, the appearance of wrinkles is visibly reduced after 14–28 d of consecutive application, however, maltose microneedle treatment delivers a higher active load to the epidermis and dermis in a shorter time that would ultimately improve the efficacy of the treatment. To further improve the deliver of M. africana or myrsinoside B to the dermis, additional techniques such as nanoencapsulation should be investigated.

## Conclusion

The findings in the current study supports the use of *M. africana* and myrsinoside B as agents capable of diminishing the aging cascade by targeting the three critical processes associated with aging skin: oxidative stress, abnormal proteolytic degradation, and chronic inflammation. Further to this, *in vitro* investigations confirm the potential of these agents to be assimilated into the skin by implementing microneedle technology, where their efficacy will be most exerted. Prospective studies should investigate the application of the crude extract and myrsinoside B against additional targets implicated in the aging cascade such as AP-1, NF-kB, TGF-β, and additional inflammatory cytokines, as well as investigate the potential of nanoparticle technology for the enhancement of drug delivery and observed efficacy *in vitro*.

## Data Availability Statement

The raw data supporting the conclusions of this manuscript will be made available by the authors, without undue reservation, to any qualified researcher.

## Ethics Statement

Human skin was procured from New York firefighters skin bank as per a memorandum of understanding with Mercer University and was reviewed by University Institutional Review Board (IRB Ref No. H0303041).

## Author Contributions

BF conducted the *in vitro* biological investigations and conducted the analysis for these results. XG, AP, and AB conducted the permeation studies and analyzed the results. NL supervised the project and provided resources for the project. All authors contributed towards and edited the final manuscript as well as provided final approval of the manuscript.

## Funding

The authors acknowledge the University of Pretoria, Mercer University, and the National Research Foundation.

## Conflict of Interest

The authors declare that the research was conducted in the absence of any commercial or financial relationships that could be construed as a potential conflict of interest.
