# Supplementary material for: In Vitro Antioxidant, Anti-Inflammatory and Skin Permeation of Myrsine africana and Its Isolated Compound Myrsinoside B
Source: Front Pharmacol. 2020 Jan 8;10:1410. doi: 10.3389/fphar.2019.01410 (PMC6960167; doi:10.3389/fphar.2019.01410)

**The *In Vitro* Antioxidant, Anti-Inflammatory and Skin Permeation of *Myrsine africana* and it’s Isolated Compound Myrsinoside B**

**Bianca Fibrich ^1^, Xinyi Gao ^2^, Ashana Puri ^2^_,_ Ajay K. Banga ^2^ and Namrita Lall ^1,3^**

^1^Department of Plant and Soil Sciences, University of Pretoria, Pretoria, South Africa

^2^ Center for Drug Delivery Research, Department of Pharmaceutical Sciences, College of Pharmacy, Mercer University, Atlanta GA, USA

^3^School of Natural Resources, University of Missouri, USA

*** Correspondence:**Namrita Lall

Namrita.lall@up.ac.za

Keywords: *Myrsine africana*_1_, myrsinoside B_2_, lipoxygenase_3_, skin delivery_4_, microneedles_5_.

**>>>Supplementary data<<<**

**Figure S1: HPLC Chromatogram of Myrsinoside B in the hydrogel**


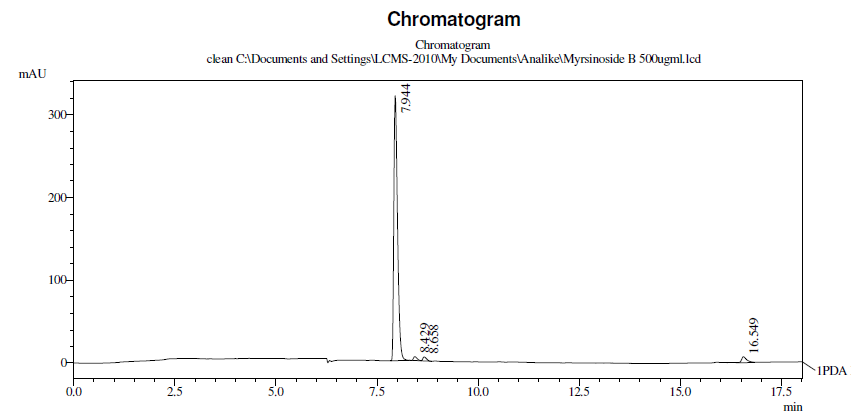


**Figure S2: LCMS Chromatogram of Myrsinoside B**


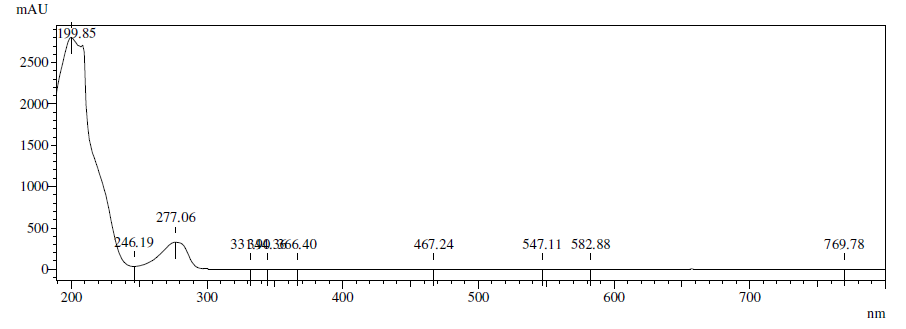
**Figure S3: UV Spectrum of Myrsinoside B**


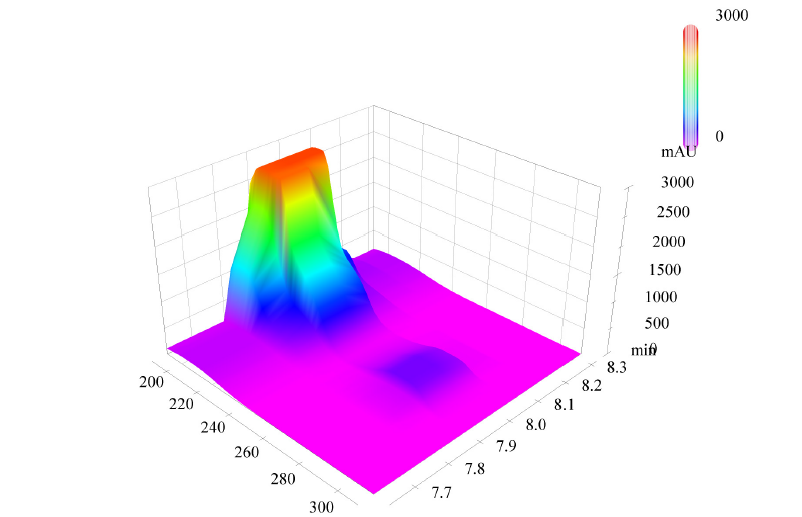


**Figure S4: 3D Graph of Myrsinoside B**

**Table S1: Peak integration of Myrsinoside B LCMS data**


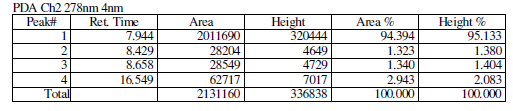

Supplement: Supplementary file 1 [file DataSheet_1.docx]
